# Serum leptin in renal transplant patients

**DOI:** 10.12861/jrip.2013.19

**Published:** 2013-06-01

**Authors:** Mahmoud Rafieian-Kopaei, Hamid Nasri

**Affiliations:** ^1^Medical Plants Research Center, Shahrekord University of Medical Sciences, Shahrekord, Iran; ^2^Department of Internal Medicine, Shahrekord University of Medical Sciences, Shahrekord, Iran

**Keywords:** Leptin, Kidney transplantation, Renal function

## Abstract

Leptin is a small peptide hormone that is mainly produced in adipose tissues. Leptin plays animportant role in regulating appetite and energy expenditure and may be involved in modulatingbone mineralization. This study was designed to test the association of serum leptin kidneyfunction in renal transplant recipients. We studied 72 kidney transplanted recipients. In thisstudy a significant difference of serum leptin between males and females with higher values infemales was seen (p>0.05). There was not relationship between serum leptin with body massindex, age and creatinine clearance (p>0.05). A negative relationship between serum leptin andthe duration of kidney transplantation was found (r= -0.26, p= 0.03). The clinical significanceof inverse association between serum leptin and the duration of renal transplantation, shouldconsider more in larger studies.

**Implication for health policy/practice/research/medical education:**

The clinical significance of inverse association between serum leptin and the duration of renal transplantation, should consider more in larger studies.

## Introduction

Renal transplantation (RT) is a treatment of choice for patients with end-stage kidney diseases. Kidney transplantation corrects most of the metabolic disorders (1). Leptin is released from fat tissues and circulating levels of leptin have been shownto be positively associated with fat mass in both lean and obeseindividuals (1-3). Previous studies have shown elevated serum leptin levels in renal failure patients (2-4). Leptin level is increased before transplantation, and decreased in the first year of RT (2-6). Increase in serum leptin level may be seen thereafter and may be due to increase in fat mass, insulin resistance or steroid use in kidney transplant patients (4-8). Leptin first emerged as a component of aregulatory fat mass and energy expenditurebut recent evidence showed that leptin might be a potentialmediator of the protective effects of fat mass on bone tissues (7-9).

## Objectives

This study was designed to test the association of serum leptin kidney function in renal transplant recipients.

## Patients and Methods

### Patients

This cross-sectional study was conducted on a group of stable kidney transplant patients, referred to the clinic of nephrology to continue their treatment. Exclusion criteria included presence of acute rejection, any active or chronic infection, taking antibiotics during the past two months, and taking medications such as non steroidal anti-inflammatory drugs. After admission, all patients were examined for body mass index. Patients' histories concerning the length of the time that they were undergone kidney transplantation and their treatments were also obtained. Body mass index (BMI) was calculated using the standard formula (weight in kilograms/height in square meters).

### Laboratory methods

Serum leptin was measured as follows; blood samples were drawn after an overnight fasting, and centrifuged within 15 minutes of drawing. The levels were measured by an enzyme-linked immunosorbent assay (ELISA) method using DRG kit (DRG diagnostic, Berline, Germany). Serum leptin normal range for males was considered to be 3.84±1.79 and for females 7.36±3.73 ng/mL. After an overnight fast peripheral venous blood samples were collected for biochemical analysis including serum creatinine, blood urea nitrogen using standard kits. Creatinine clearance was evaluated from serum creatinine, considering age and body weight (9).

### Ethical issues

(1) The research followed the tenets of the Declaration of Helsinki; (2) informed consent was obtained; (3) the research was approved by ethical committee of Shahrekord University of Medical Sciences.

### Statistical Analysis

The results were expressed as the mean±SD and median values. A statistical correlation was assessed using a partial correlation test. Comparison between groups were done using student’s *t-* test. For normalization of leptin data, their second square were used. All statistical analyses were performed with the SPSS 11.5 (SPSS Inc., Chicago, USA). Statistical significance was determined at a p< 0.05.

## Results

A total of 72 patients were enrolled in this study, including 47 men, 25 women, in whom, 11 patients had diabetes mellitus. The mean patients’ age was 44±12 years. The transplantation period was 67.5±42 months (median: 62 years). The patients’ data are shown in Table 1. In this study there was a significant difference of serum leptin between females and males (p< 0.001). There was no significant difference of serum leptin between diabetic and non-diabetic patients. In this study, there was an inverse association between serum leptin and duration of renal transplantation (r=-0.26, p= 0.03). There was not any significant association between serum leptin and creatinine clearance, BMI or ages of the patients (P> 0.05).

**Table 1:** Data of the patient

**N=72**	**Minimum**	**Maximum**	**Mean + SD**	**Median**
Age (year)	13	61	44 ± 12	44
DKT (month)*	4	162	67.5 ± 42	62
BMI (kg/m^2^)	15	33	24 ± 4	17
*duration of kidney transplantation, **Intact PTH (iPTH)				

## Discussion

In the current study we found a significant difference of serum leptin between females and males, while there was no significant difference of serum leptin between diabetic and non-diabetic patients. There was an inverse association between serum leptin and duration of renal transplantation. There were not any significant association between serum leptin and creatinine clearance or age of the patients.

Souza *et al*. carried out a study on thirty-two RT patients and found no correlation between leptin and renal function in their study. They showed also that during the first post transplant year, serum leptin levels decreased significantly in relation to pre-transplant period (10).

Nicoletto *et al*. studied thirty-two kidney transplant recipients ,who were followed up for 5 years after transplantation. They found leptin level decrease in the immediate post-transplant period and remain reduced for at least one year. Five years after transplantation, leptin have a profile similar to those in the pre-transplant period. They concluded that leptin profile is possibly associated with the elevated incidence of cardiovascular diseases observed in the late post-transplant period (11).

## Conclusion

The clinical significance of inverse association between serum leptin and the duration of renal transplantation, should consider more in larger studies.

## Authors' Contributions

HN defined the aims of research. HN prepared the paper. MRK edited the manuscript.

## Conflict of Interests

The authors declare that they have no conflict of interest.

## Ethical considerations

Ethical issues (including plagiarism, data fabrication, double publication) have been completely observed by the author.

## Funding/Support

This study was supported by a grant from Shahrekord University of Medical Sciences.
